# Patient Characteristics Associated With Annual Nutrition Visits in Children With Type 1 Diabetes

**DOI:** 10.1155/pedi/4108685

**Published:** 2025-03-28

**Authors:** Svetlana Azova, Belinda S. Lennerz, Carter R. Petty, Erin Gordon, Hannah Michelson, Anna Schmidt, Katharine Garvey, Erinn T. Rhodes

**Affiliations:** 1 Division of Endocrinology, Boston Children’s Hospital, 300 Longwood Avenue, Boston 02115, Massachusetts, USA; 2 Department of Pediatrics, Harvard Medical School, 25 Shattuck Street, Boston 02115, Massachusetts, USA; 3 Biostatistics and Research Design Center, Boston Children’s Hospital, 300 Longwood Avenue, Boston 02115, Massachusetts, USA; 4 Division of Gastroenterology, Hepatology and Nutrition, Boston Children’s Hospital, 300 Longwood Avenue, Boston 02115, Massachusetts, USA

**Keywords:** healthcare utilization, medical nutrition therapy, patient care, pediatric, registered dietitian, type 1 diabetes

## Abstract

**Objective:**

Diabetes organizations recommend nutrition education by a registered dietitian (RD) at least annually following type 1 diabetes (T1D) diagnosis in children. The study objectives were to describe differences over time in annual RD follow-up in children with T1D and to identify patient characteristics associated with RD engagement.

**Research Design and Methods:**

Data based on 6034 completed diabetes medical visits among 1982 patients aged <18 years with T1D for ≥1 year followed at a pediatric, tertiary care, academic medical center over a 5-year period (2018–2022) were analyzed. Generalized estimating equations models assessed for differences over time in the rates of RD visit completion in the year preceding the last diabetes medical encounter and identified sociodemographic, diabetes care-related, and clinical patient characteristics associated with RD follow-up. Models were fit for the whole sample and groups subset by race and ethnicity.

**Results:**

Observed annual RD follow-up rate over the 5-year period was 20.8%, with the lowest adjusted percentage in 2021 compared to 2018. In multivariable analysis, for each year increase in age (*p* = 0.004) and diabetes duration (*p*<0.001), there was a 3% and 15% reduction in the odds of RD follow-up, respectively. RD follow-up was associated with lower hemoglobin A1c within the subsequent year in adjusted analysis (*p* = 0.029), with the greatest improvement among Hispanic patients.

**Conclusions:**

Annual RD visit frequency among children with T1D is suboptimal. Study findings provide insights for targeted intervention to improve RD engagement. RD follow-up may be associated with improved glycemic outcomes.

## 1. Introduction

Medical nutrition therapy (MNT) is a pillar of diabetes care. It supports the primary goals in pediatric diabetes management to achieve optimal metabolic control and to reduce the risk of micro- and macrovascular complications, while preserving normal growth and development. The American Diabetes Association (ADA) and International Society for Pediatric and Adolescent Diabetes both recognize the importance of individualized MNT in children with type 1 diabetes (T1D) and emphasize the need for comprehensive nutrition education by an experienced registered dietitian (RD) at diagnosis, followed by more frequent visits within the first year and annually thereafter [1, 2]. Studies show improved glycemic control [3–6] and other positive metabolic outcomes, such as maintenance of a normal body weight [7, 8] and improvement in the lipid profile [9], with attention to nutritional considerations. However, many families struggle to meet the currently recommended benchmarks for both RD follow-up [10–12] and a healthful diet [6, 13–19]. Inadequate utilization of MNT may contribute to negative health outcomes in children with T1D, including challenges with meeting glycemic targets [20] and high rates of overweight/obesity [21–26].

At Boston Children’s Hospital (BCH), follow-up with an outpatient RD is scheduled at the time of the T1D diagnosis and then encouraged annually thereafter. RD visits are scheduled separately from the quarterly medical visits. Longitudinal data monitored by the BCH Endocrinology Program suggested suboptimal RD follow-up among established patients with T1D. However, to our knowledge, factors associated with RD engagement had not been previously investigated in a systematic manner. Understanding these issues is a critical first step in recognizing potential barriers to and inequities in MNT utilization and helping to inform future studies aimed at exploring strategies to improve attention to nutritional recommendations in children with T1D. This is especially important in the era of increased uptake of technologies, including hybrid closed-loop (HCL) systems, which may result in improved overall glycemic control but still present with ongoing challenges with postprandial glycemic excursions and weight gain [27].

In this retrospective study, we aimed to (1) describe differences over time in the percentage of established pediatric patients with T1D who have had ≥1 RD visit in the preceding year, (2) identify sociodemographic and diabetes care-related factors associated with RD engagement, and (3) assess the association between RD visits and clinical characteristics of the child within the subsequent year. The goal is to provide preliminary data to drive the design of future studies aimed at optimizing the uptake of MNT in pediatric patients with T1D.

## 2. Materials and Methods

### 2.1. Study Design, Setting, and Selection of Participants.

This was a retrospective study of children aged 1–17 years with T1D followed by the Diabetes Program at BCH, a large, pediatric, tertiary care, academic medical center, during a 5-year period between January 1, 2018 and December 31, 2022. This Diabetes Program manages ~250 cases of new-onset diabetes per year and has a population of ~2200 total patients with diabetes. It is a major referral site for patients with diabetes from multiple states, including Massachusetts, New Hampshire, Rhode Island, Maine, and Vermont. The outpatient Diabetes Program is comprised of a multidisciplinary team of providers, including ~40 endocrinologists (31 attending physicians and 9 fellows), 11 diabetes nurse educators (DNEs), 9 RDs, and 3 social workers.

Data were arranged in yearly cohorts from 2018 to 2022. Within each cohort, we excluded patients with non-T1D and diabetes duration <1 year, as well as those who transferred into the BCH Diabetes Program less than a year prior to their last endocrinologist/DNE encounter in that calendar year and those who do not receive their regular diabetes care at BCH (including patients seen for second opinions, consultations for non-diabetes concerns, and international patients who only have periodic diabetes follow-up at BCH). Ethics approval was granted by the BCH Institutional Review Board. A waiver of informed consent was obtained.

### 2.2. Variables of Interest.

Eligible patients with T1D were identified from an Outpatient Diabetes Population Management database used for quality improvement, as well as review of the electronic medical record (EMR). For each calendar year, data were collected from the last endocrinologist/DNE encounter (excluding visits exclusively for continuous glucose monitor [CGM] or pump initiation/transition) in the BCH Diabetes Program, up to 18 years of age, whichever was sooner. For each year, patients were grouped into those who had an RD visit within 1 year preceding their last endocrinologist/DNE encounter and those who did not.

Sociodemographic variables collected included date of birth, sex, race and ethnicity, primary language, need for interpreter, primary insurance type, and zip code, as documented in the EMR. For race and ethnicity, we included categories for (1) White, non-Hispanic, (2) Black, non-Hispanic, (3) Hispanic, (4) Asian, non-Hispanic, (5) Multiracial, non-Hispanic, and (6) Another, non-Hispanic (as documented in the EMR). Those whose race and ethnicity were listed as “Unknown” or “Declined to Answer” (3.2% of total cases) were classified as “unknown” and excluded from the analyses. Primary language was categorized as (1) English, (2) Spanish, or (3) other. Primary insurance type was categorized as either private or public using definitions applied by the BCH EMR (those without insurance were classified under public insurance). Zip codes were linked to neighborhood income and educational status using American Community Survey (ACS) data for that calendar year [28, 29]. Specifically, percentage of families in the respective zip codes who had incomes below 200% of the federal poverty level (FPL), considered low income [30], and of families with householders who had completed less than a college degree were obtained.

Diabetes care-related variables at the last endocrinologist/DNE encounter for each calendar year included the date of the most recent visit with an RD that preceded the encounter; diabetes duration, defined as the time between the date when the diagnosis code for “type 1 diabetes” (ICD-9 codes 250.*X*1 or 250.*X*3, where *X* = 0–9, or ICD-10 codes E10.*X*) first appeared in the EMR (confirmed/updated based on EMR information) and the date of the encounter; use of a CGM; and mode of insulin delivery (multiple daily injections, insulin pump without automated insulin delivery, or HCL system).

Additional clinical variables at the last endocrinologist/DNE encounter for each calendar year included hemoglobin A1c (HbA1c) and body mass index (BMI) *z*-score. For missing data, we performed an EMR review to find the most proximal value, provided that it was within 1 year preceding the last endocrinologist/DNE encounter. If both point-of-care testing and whole blood HbA1c results were available on the same day, we included the whole blood HbA1c value.

### 2.3. Statistical Analyses.

The percentage of patients with ≥1 RD visit(s) in the preceding year was calculated for each year between 2018 and 2022 to compute the observed annual RD follow-up rate over the 5-year period. Summary statistics for each year were presented as observed means and standard deviations or percentages, as appropriate. Generalized estimating equations (GEE) with robust standard errors (SEs) clustering at the patient level were conducted to assess for differences over time in CGM use and mode of insulin delivery (controlling for age, sex, race and ethnicity, and diabetes duration) and HbA1c and BMI *z*-score within the subsequent year (controlling for age, sex, diabetes duration, race and ethnicity, CGM use, and mode of insulin delivery). Models were fit for the whole sample as well as for groups subset by race and ethnicity categorized as either (1) White, non-Hispanic, (2) Black, non-Hispanic, or (3) Hispanic. We chose these groups as they were the most common ones followed by the BCH Diabetes Program, comprising 92% of the sample.

For zip-code matched data, medians and interquartile ranges were calculated for the percentage of families in the respective zip codes who had incomes below 200% of the FPL and of those with householders who had completed less than a college degree in each calendar year. Patients were categorized as living in low-income zip codes if the percentage of households in those zip codes was in the upper quartile (i.e., above the 75th percentile) for 200% below the FPL. Patients were categorized as living in zip codes with lower educational attainment if the percentage of householders in those zip codes was in the upper quartile for having completed less than a college degree.

GEE with robust SEs clustering at the patient level was conducted to assess for differences in the rates of RD visit completion in the year preceding the last endocrinologist/DNE encounter in years 2019–2022 compared with 2018, adjusted for repeated measures, and to identify potential sociodemographic and diabetes care-related characteristics associated with RD visits. Both bivariate and multivariable analyses were conducted for the latter and included calendar year as one of the variables to account for potential time-related confounding. To assess the relationship between RD visits and clinical variables (HbA1c and BMI *z*-score within the subsequent year), GEE with robust SEs clustering at the patient level was conducted, with adjustment for age, sex, race and ethnicity, diabetes duration, CGM use, and mode of insulin delivery. Models were fit for the whole sample as well as for groups subset by race and ethnicity, as per above. A two-sided *p*-value of <0.05 was used to define statistical significance. Stata Special Edition software package (Version 16.1, StataCorp LLC, College Station, TX, USA) was used for statistical analyses.

## 3. Results and Discussion

### 3.1. Results.

A total of 6034 observations among 1982 unique patients followed by the BCH Diabetes Program between 2018 and 2022 were included in the analysis. Sociodemographic, diabetes care-related, and clinical patient characteristics by year for the whole sample are summarized in Table 1. The observed percentage of patients who used a CGM increased across all 5 years, with the greatest increase from 2018 to 2019 (52.3%–71.3%) and plateauing by 2021–2022 (84.7%–89.9%). Use of an HCL system increased steadily after 2019, with the largest increase from 2021 to 2022 (17.9%–36.8%). There was a decline in HbA1c (years 2021 and 2022 differed significantly from 2018) and a rise in BMI *z*-score (years 2019, 2021, and 2022 differed significantly from 2018) in adjusted analyses.

**Figure 1:**
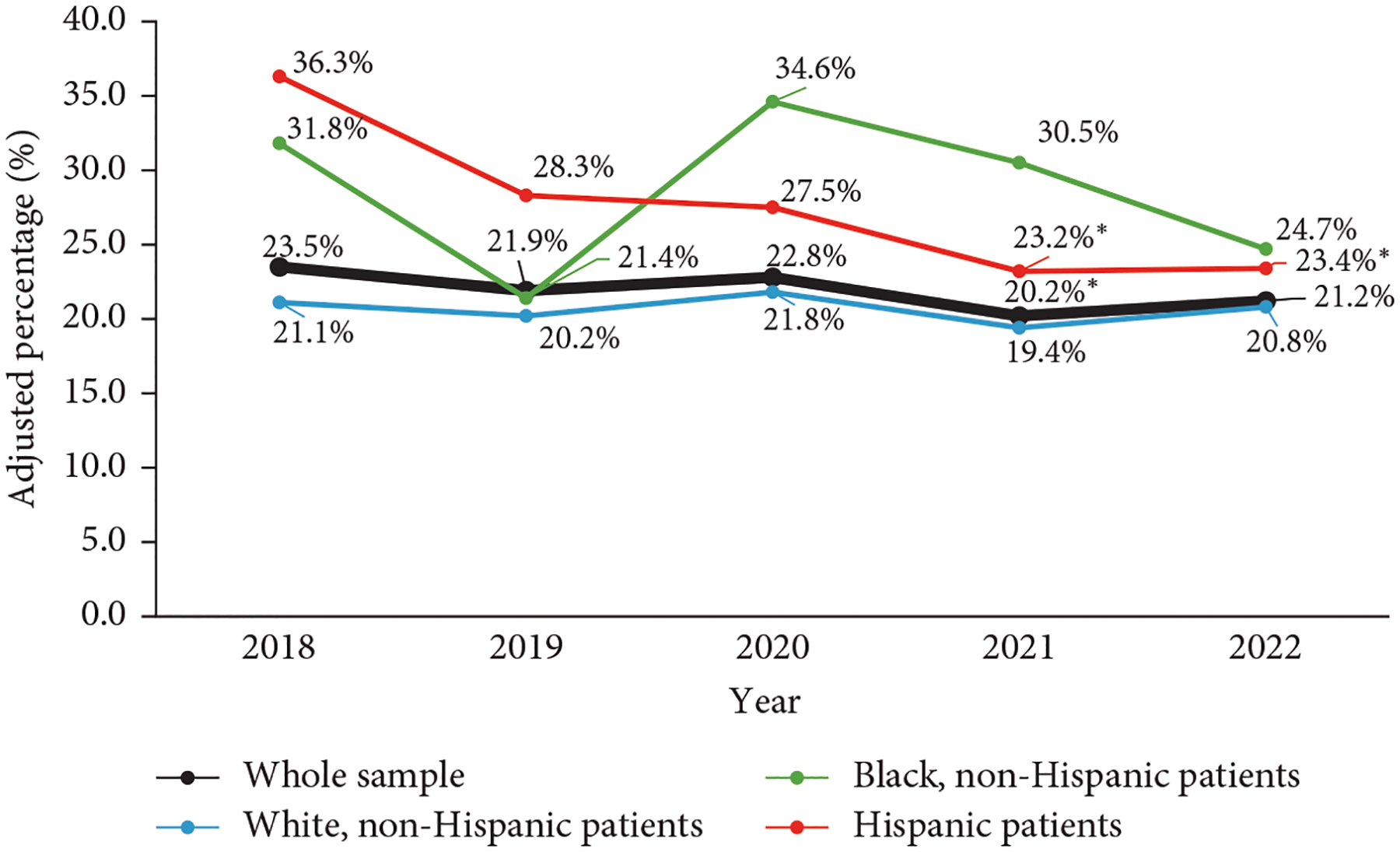
Adjusted annual percentages of established pediatric patients with type 1 diabetes with ≥1 registered dietitian visit in the preceding year between 2018 and 2022. Data are presented for the whole sample and when subset by race and ethnicity. *Values in these years differed significantly from 2018, *p*<0.05.

The observed annual RD follow-up rate over the 5-year period for the whole sample was 20.8%. Adjusted percentages over time for the whole sample and when subset by race and ethnicity are presented in Figure 1. For the whole sample, there was a significantly lower follow-up in 2021 compared to 2018 (odds ratio [OR] 0.82 [95% confidence interval 0.69–0.98; *p* = 0.027]) in analysis adjusted for repeated measures. In bivariate analyses, higher likelihood of RD follow-up was associated with younger age (OR 0.90 [0.88–0.92; *p*<0.001]), race and ethnicity (OR 1.55 [1.10–2.17; *p* = 0.012] for Black, non-Hispanic patients; OR 1.44 [1.12–1.85; *p* = 0.004] for Hispanic patients), Spanish language (OR 2.56 [1.63–4.04; *p*<0.001]), need for interpreter (OR 1.87 [1.23–2.86; *p* = 0.004]), public insurance (OR 1.32 [1.12–1.55; *p* = 0.001]), living in low-income neighborhoods (OR 1.19 [1.01–1.41; *p* = 0.042]), shorter diabetes duration (OR 0.83 [0.80–0.86; *p*<0.001]), and the use of CGM (OR 1.18 [1.01–1.39; *p* = 0.038]), while lower likelihood was observed with the use of insulin pump (OR 0.75 [0.64–0.87; *p*<0.001]) and HCL system (OR 0.69 [0.55–0.86; *p* = 0.001]) (Table 2). In multivariable analysis, younger age (OR 0.97 [0.94–0.99; *p* = 0.004]) and shorter diabetes duration (OR 0.85 [0.82–0.88; *p*<0.001]) remained predictive of RD follow-up (Table 3). For each year increase in age and diabetes duration, there was a 3% and 15% reduction in the odds of RD follow-up, respectively. RD visits were associated with a 0.09% or 0.93 mmol/mol reduction (−0.16 to −0.01% or −1.77 to −0.09 mmol/mol, respectively; *p* = 0.029) in HbA1c within the subsequent year when adjusted for age, sex, race and ethnicity, diabetes duration, CGM use, and mode of insulin delivery. There was no association between RD visits and BMI *z*-score within the subsequent year (0.01 [−0.02 to 0.04; *p* = 0.512]).

In analyses subset by race and ethnicity, the observed annual RD follow-up rates for (1) White, non-Hispanic, (2) Black, non-Hispanic, and (3) Hispanic patients over the 5-year period were 19.5%, 27.4%, and 26.8%, respectively. There were no statistically significant differences between the years in analyses adjusted for repeated measures for White, non-Hispanic (*p* = 0.533), and Black, non-Hispanic (*p* = 0.336) patients. For Hispanic patients, in analysis adjusted for repeated measures, there was significantly lower follow-up in 2021 (OR 0.53 [0.31–0.90; *p* = 0.019]) and 2022 (OR 0.54 [0.31–0.92; *p* = 0.025]) compared to 2018. Detailed sociodemographic, diabetes care-related, and clinical patient characteristics by year subset by race and ethnicity are summarized in Supporting Information 1: Table S1.

Among White, non-Hispanic patients (Supporting Information 2: Table S2a), in multivariable analysis, higher likelihood of RD follow-up was associated with younger age (OR 0.96 [0.94–0.99; *p* = 0.004]) and shorter diabetes duration (OR 0.85 [0.82–0.89; *p*<0.001]). In this group, RD follow-up was also associated with a 0.09% or 0.96 mmol/mol reduction (−0.17 to −0.00% or −1.87 to −0.05 mmol/mol, respectively; *p* = 0.038) in HbA1c within the subsequent year in adjusted analysis. Among Black, non-Hispanic patients (Supporting Information 2: Table S2b), in multivariable analysis, higher likelihood of RD follow-up was only associated with shorter diabetes duration (OR 0.81 [0.66–0.98; *p* = 0.041]). Among Hispanic patients (Supporting Information 2: S2c), in multivariable analysis, RD follow-up was associated with need for interpreter (OR 2.93 [1.01–8.54; *p* = 0.048]), living in zip codes with lower educational attainment (OR 1.77 [1.00–3.13; *p* = 0.049]), and shorter diabetes duration (OR 0.87 [0.80–0.95; *p* = 0.003]). RD follow-up was also associated with a 0.27% or 2.95 mmol/mol reduction (−0.52% to −0.02% or −5.67 to −0.23 mmol/mol, respectively; *p* = 0.034) in HbA1c in this cohort.

### 3.2. Discussion.

To our knowledge, this is among the largest single-center studies that systematically examined objective healthcare data on nutrition care among established pediatric patients with T1D, including differences over time in RD follow-up rates and factors and potential clinical outcomes associated with RD engagement. The study found suboptimal RD follow-up rates among established patients with T1D followed in a pediatric, tertiary care, academic medical center. These rates were lower than those previously reported in other studies; however, those studies relied on survey responses [10, 11], and one was based on international data [11]. A recent cross-sectional study that analyzed national claims data for youth with diabetes in the United States found that only 11.8% of patients with T1D had a claim with a dietitian over about a 1-year period [12]. This may suggest that RD follow-up among patients with T1D is suboptimal on a national level. Alternatively, this may also suggest that claims for RD visits are not being consistently filed [12]. In our study, in multivariable analysis, a higher likelihood of RD visits was associated with younger age and shorter diabetes duration. RD visits were associated with a statistically significant decrease in HbA1c within the subsequent year in adjusted analysis, although the clinical significance of this improvement in HbA1c may be marginal (a decrease of 0.09% or 0.93 mmol/mol).

A recent survey assessing nutrition experiences among caregivers of youth under age 18 years with T1D for ≥1 year who receive their diabetes care at BCH found that only 56.6% of respondents considered it necessary to see an RD every year [31]. This percentage was notably higher than the actual follow-up rates in our study (20.8% observed annual follow-up rate over the 5-year period), suggesting that caregiver beliefs and intentions may not always align with actual practices or that there may be barriers to accessing care. In the survey, older age and shorter duration of diagnosis were associated with the caregiver belief that it is necessary to see an RD yearly. Interestingly, while the association with shorter diabetes duration was concordant with our objective findings, we found younger rather than older patient age to be predictive of higher likelihood of RD visits. One potential explanation is that caregivers of older patients may have stronger views about the necessity for annual RD visits, but this may not translate into successful follow-up.

Healthcare utilization patterns seen in other medical settings also show that adolescents have lower than expected rates of annual preventive primary care visits [32–35] and subspecialty follow-up [36]. In contrast, younger patients are more dependent on caregiver decisions and actions, and caregivers are generally more proactive in managing their child’s healthcare needs during early childhood, resulting in better adherence with medical follow-up [37]. This may at least partially explain our finding of younger age being predictive of higher likelihood of RD visits. In addition, shorter disease duration may be associated with overall better adherence with health-related recommendations, as seen in other pediatric disease states [38–41]. In the context of RD follow-up among pediatric patients with T1D, this observation may be due to a number of a factors, including overall heightened caregiver concern and involvement earlier on in the child’s diagnosis, the perception of limited knowledge and the desire for guidance and new information, and increased initial emphasis on close follow-up by other members of the diabetes care team. Conversely, increased self-perceived knowledge and comfort with nutritional topics may be contributing to lower RD follow-up rates with longer diabetes duration, especially among adolescents with evolving diabetes self-management skills.

The above findings highlight the need for future interventions aimed at increasing the utilization of RD services, with specific emphasis on older patients and those with longer standing diabetes duration. Decreasing MNT utilization over time and with increasing age can result in missed opportunities for optimization of dietary behaviors and associated prandial diabetes management strategies. This may in turn negatively impact patients’ glycemic control and increase future risk of other health complications. Therefore, refreshment and routine reinforcement of healthy nutritional habits is vital, especially at times of major transitions, such as emerging independence during adolescence. However, as children with diabetes rely on caregiver choices and perceptions when it comes to all aspects of care, including nutrition [42, 43], even as they age, initiatives targeting both patients and caregivers are essential.

Our study also found an association between RD visits and a lower HbA1c within the subsequent year. Although the improvement in HbA1c was marginal, statistical significance persisted even when the analysis was adjusted for age, sex, race and ethnicity, diabetes duration, CGM use, and mode of insulin delivery. This has several potential clinical and public health implications. Despite vast pharmacological and technological advances, recent data from the T1D Exchange Quality Improvement Collaborative showed that only 25.6% of youth are currently meeting the ADA HbA1c goal of <7% for the prevention of long-term complications [44]. Although likely multifactorial, suboptimal attention to nutritional considerations and underutilization of MNT, including RD services, may be contributing to those findings. This is especially important in the era of increased use of diabetes technologies. Use of CGM and HCL systems had increased significantly over the 5-year period in our sample, and both have been associated with improvements in glycemic control [20, 45, 46]. However, despite improvements in overall glycemic outcomes, challenges with postprandial excursions will likely persist, especially if patients loosen their dietary practices and prandial diabetes management strategies [47, 48]. This is important to consider as reliance on and uptake of these advanced diabetes technologies continues to increase, while the rates of RD follow-up, at least in this study, are not going up concurrently, potentially limiting opportunities for further glycemic optimization. In addition, although there was no association between RD visits and BMI *z*-score within the subsequent year, the gradual increase in the BMI *z*-score during our study period, starting even before the COVID-19 pandemic, a finding corroborated by other recent literature [44], is notable, suggesting that patients will likely benefit from nutritional counseling regardless. Thus, MNT needs to be recognized as an important complementary component of diabetes care.

When subset by race and ethnicity, there was a statistically significant decrease in HbA1c among White, non-Hispanic and Hispanic patients but not among Black, non-Hispanic patients. Barriers to incorporating the recommended dietary changes, such as potential lack of access to the necessary resources and presence of food insecurity (a high percentage of Black, non-Hispanic patients lived in low-income zip codes), may have contributed to the absence of glycemic improvement among Black, non-Hispanic patients. However, it is important to note that in our study, Black, non-Hispanic and Hispanic patients with T1D represented only a small proportion of the total population (4%–5% and 9%–11%, respectively), and the relatively smaller sample sizes could have affected our findings. However, it is notable that Hispanic patients had the most clinically meaningful decrease in their HbA1c compared to the whole sample and White, non-Hispanic patients, despite the smaller sample size. Our colleagues recently described culturally based nutritional challenges in a qualitative analysis of Hispanic caregivers’ experience with T1D at BCH [49]. Preliminary insights from this study, which was conducted between 2019 and 2020, may have led to increased awareness of these reported challenges and impacted subsequent RD practice at BCH by highlighting the need for tailored counseling. This may have contributed to dietary changes and the glycemic improvement observed in Hispanic patients in our study. These findings also raise interesting questions about the potential role that baseline culturally based differences in nutritional quality and dietary composition may play in facilitating the successful implementation of RD advice. Furthermore, among Hispanic patients, there were associations between RD follow-up and both need for interpreter and living in zip codes with lower educational attainment. This suggests a potential association between certain family-level sociodemographic factors and recognized need for additional nutrition education. The role of culturally based integration of provider advice and counseling, as well as insights about nutritional quality and dietary composition, should be explored in future studies.

An additional observation of interest was the effect of the COVID-19 pandemic on the rates of RD follow-up overall and when subset by race and ethnicity, which our study facilitated by including both pre-pandemic (2018–2019) and post-pandemic (2020–2022) periods. Overall, significantly lower RD follow-up was observed in 2021 compared to 2018. While there were no differences in the rates of RD follow-up by year among White, non-Hispanic and Black, non-Hispanic patients, Hispanic patients had significantly lower RD follow-up in both 2021 and 2022 compared to 2018. While reasons for this are unclear, it is important to note that during and post-pandemic, almost all RD visits at BCH were converted to telehealth appointments. Lower utilization of telehealth visits has previously been described in historically marginalized populations, both before and during the COVID-19 pandemic [50, 51]. It is possible that Hispanic patients in our study may have encountered technological or other barriers in attending telehealth visits post-pandemic, as described in other studies [52]. This is important to investigate further to ensure that inequities in the utilization of telehealth appointments are addressed promptly and appropriately, especially as these visit types continue to be a staple of clinical practice for nutrition services.

This study has several limitations. One limitation is the lack of generalizability. Although BCH is a large, pediatric, academic medical center that serves a diverse population of children with diabetes across multiple states, our sample may not be representative of all youth with diabetes on a national level. For example, our center serves a population of patients with T1D that is predominantly White, non-Hispanic (75%–80%), a slightly higher proportion than that observed in the T1D Exchange Quality Improvement Collaborative [53]. In addition, there is variation in nutrition education practices among diabetes centers [11], and different institutions have variable resources and capabilities to provide multidisciplinary care to patients, which may impact engagement with RD visits in ways that are not encountered by our program. To further assess trends in and barriers to RD follow-up among patients with T1D, large-scale, national collaborations should be pursued. One area of interest worth investigating further is whether centers that combine RD visits on the same day as medical visits with an endocrinologist or DNE in a standard fashion, which our institution does not routinely do, have higher RD follow-up rates [12]. If so, this may be a target of future interventional studies.

Another limitation of this retrospective study is that it does not allow us to investigate the reasons for suboptimal RD follow-up rates in our population or explain the discrepant findings by race and ethnicity. The previously mentioned survey study that assessed caregiver experiences with RD visits, which included mostly White, non-Hispanic participants (94%), showed that 47.8% of caregivers reported satisfaction with their nutrition care, with shorter duration of diagnosis being associated with a higher likelihood of satisfaction [31]. Respondents identified several reasons for their lack of RD follow-up in the preceding 12 months, including perceived sufficient knowledge in nutrition and suboptimal past RD experiences. In addition, only 61.4% reported encouragement from an endocrinologist or DNE to meet with an RD. Prior studies suggested that despite being able to recognize the general concepts associated with healthful eating, caregivers of children with T1D identified several barriers to its implementation [54, 55]. These included time constraints, expense, child food preferences, and picky eating [54], as well as themes unique to diabetes, such as caregiver desire to avoid limiting their child’s diet or making them “feel different” [54] and difficulties associated with carbohydrate estimation [55]. It is unclear if these factors may have affected caregiver willingness to follow up with an RD. To further assess both caregiver and patient perceptions, experiences, and barriers when it comes to MNT utilization in T1D, our team is currently conducting a mixed-methods research study.

Preliminary studies investigating barriers to RD utilization in children with T1D are an important prerequisite to designing or adapting interventions aimed at increasing MNT uptake. Previous research on this topic has identified several nutrition-based interventional strategies that may be associated with positive effects on diet quality and/or glycemic or cardiometabolic outcomes [56]. These strategies have included general [57–59] and problem-solving [57] education, family-centered behavioral nutritional interventions [60, 61], and interactive methods (e.g., quizzes and multimedia applications for carbohydrate counting) [62]. Combined with our preliminary data, these approaches present potential opportunities on which to model the design of future interventions that can help improve engagement with RD services in pediatric T1D.

## 4. Conclusions

In conclusion, our study showed suboptimal longitudinal RD follow-up rates among established patients with T1D at a large, pediatric, academic medical center and identified several factors and outcomes associated with a higher likelihood of RD engagement. Future investigations utilizing mixed methods are needed to explore caregiver and patient perceptions, experiences, and barriers regarding MNT. These studies may provide more context for the objective findings identified by this study, including potential differences by race and ethnicity. Ultimately, insights from this research will be used to design interventions aimed at increasing the uptake of MNT in pediatric patients with T1D. This is especially important in the era of increased reliance on advanced diabetes technologies, which may not eliminate all challenges with postprandial glycemic excursions or other health risks, such as the development of overweight and obesity and their associated complications.

## Supplementary Material

Supplemental Table S1 *Supporting Information 1*. Table S1 summarizes detailed sociodemographic, diabetes care-related, and clinical characteristics of children with type 1 diabetes by year subset by race and ethnicity.

Supplemental Table S2 *Supporting Information 2*. Table S2 summarizes associations between patient characteristics and annual visits with a registered dietitian in children with type 1 diabetes subset by race and ethnicity (Table S2a: White, non-Hispanic patients; Table S2b: Black, non-Hispanic patients; and Table S2c: Hispanic patients).

## Figures and Tables

**Table 1:** Characteristics of established pediatric patients with type 1 diabetes by year.

	Data by year (observed mean ± SD or percentage)				
Characteristic	2018 (*n* = 1206)	2019 (*n* = 1193)	2020 (*n* = 1181)	2021 (*n* = 1226)	2022 (*n* = 1228)
Sododemographic characteristics					
Age (years)	13.7 ± 3.5	13.7 ± 3.6	13.6 ± 3.7	13.5 ± 3.7	13.4 ± 3.7
Female sex	48.8%	48.6%	50.0%	49.3%	47.3%
Race and ethnicity*^,†^					
White, non-Hispanic	79.7%	78.8%	77.9%	77.1%	76.3%
Black, non-Hispanic	4.4%	4.4%	4.0%	4.9%	4.8%
Hispanic	9.0%	9.6%	9.9%	10.0%	10.7%
Asian, non-Hispanic	1.4%	1.3%	1.4%	1.4%	1.4%
Multiracial, non-Hispanic	1.2%	1.5%	1.7%	1.8%	2.1%
Another, non-Hispanic	4.4%	4.4%	5.1%	4.9%	4.8%
Primary language					
English	96.6%	96.6%	96.0%	95.7%	95.6%
Spanish	1.7%	1.8%	2.1%	2.2%	2.3%
Other	1.7%	1.6%	1.9%	2.1%	2.1%
Need for interpreter	2.4%	2.8%	3.0%	3.2%	3.5%
Public insurance	23.6%	24.0%	24.8%	26.9%	28.8%
Living in low-income zip codes*	24.5%	25.1%	24.7%	25.1%	24.5%
Living in zip codes with lower educational attainment*	25.1%	24.7%	25.0%	25.1%	25.6%
Diabetes care-related characteristics					
Diabetes duration (years)	6.0 ± 3.7	5.9 ± 3.6	5.7 ± 3.7	5.6 ± 3.6	5.5 ± 3.4
CGM use^‡^	52.3%	71.3%^§^	78.4%^§^	84.7%^§^	89.9%^§^
Mode of insulin delivery^†,‡^					
MDI	38.9%	38.2%^§^	39.7%	41.0%	36.9%^§^
Insulin pump	58.0%	58.4%	48.4%^§^	41.0%^§^	26.3%^§^
HCL system	3.1%	3.4%	11.9%^§^	17.9%^§^	36.8%^§^
Clinical characteristics					
HbAlc (%)^∥^	8.5 ± 1.5	8.3 ± 1.6	8.2 ± 1.6	7.9 ± 1.6^§^	7.8 ± 1.6^§^
(mmol/mol)	(68.9 ± 16.1)	(66.9 ± 17.5)	(66.5 ± 17.4)	(63.2 ± 17.8)	(61.3 ± 17.6)
BMI *z*-score^∥^	0.63 ± 0.94	0.65 ± 0.95^§^	0.69 ± 0.95	0.70 ± 0.95^§^	0.71 ± 0.96^§^

Abbreviations: BMI, body mass index; CGM, continuous glucose monitor; HbA1c, hemoglobin A1c; HCL, hybrid closed-loop; MDI, multiple daily injections; SD, standard deviation.

* Unknown data were not included in the denominator.

† Totals may not sum up to 100% due to rounding.

‡ Although observed values are reported, comparative analyses across years were adjusted for repeated measures and controlled for age, sex, race and ethnicity, and diabetes duration.

§ Values in these years differed significantly from 2018, *p*<0.05.

∥ Although observed values are reported, comparative analyses across years were adjusted for repeated measures and controlled for age, sex, race and ethnicity, diabetes duration, CGM use, and mode of insulin delivery.

**Table 2:** Bivariate analyses of associations between patient characteristics and annual visits with a registered dietitian in children with type 1 diabetes.

Characteristic	OR [95% CI]	*p*-Value
Sociodemographic characteristics		
Age (years)	0.90 [0.88–0.92]	**<0.001**
Sex		
Male	Ref	Ref
Female	0.94 [0.81–1.10]	0.435
Race and ethnicity		
White, non-Hispanic	Ref	Ref
Black, non-Hispanic	1.55 [1.10–2.17]	**0.012**
Hispanic	1.44 [1.12–1.85]	**0.004**
Asian, non-Hispanic	1.11 [0.60–2.06]	0.747
Multiracial, non-Hispanic	1.25 [0.70–2.22]	0.456
Another, non-Hispanic	1.22 [0.89–1.66]	0.210
Primary language		
English	Ref	Ref
Spanish	2.56 [1.63–4.04]	**<0.001**
Other	0.88 [0.50–1.56]	0.662
Need for interpreter		
No	Ref	Ref
Yes	1.87 [1.23–2.86]	**0.004**
Insurance type		
Private	Ref	Ref
Public	1.32 [1.12–1.55]	**0.001**
Living in low-income zip codes		
No	Ref	Ref
Yes	1.19 [1.01–1.41]	**0.042**
Living in zip codes with lower educational attainment		
No	Ref	Ref
Yes	1.11 [0.93–1.32]	0.259
Diabetes care-related characteristics		
Diabetes duration (years)	0.83 [0.80–0.86]	**<0.001**
CGM use		
No	Ref	Ref
Yes	1.18 [1.01–1.39]	**0.038**
Mode of insulin delivery		
MDI	Ref	Ref
Insulin pump	0.75 [0.64–0.87]	**<0.001**
HCL system	0.69 [0.55–0.86]	**0.001**
Time variable		
Calendar year		
2018	Ref	Ref
2019	0.91 [0.78–1.06]	0.228
2020	0.96 [0.81–1.13]	0.613
2021	0.82 [0.69–0.98]	**0.027**
2022	0.87 [0.73–1.05]	0.139

*Note*. Bold values are statistically significant associations, *p*<0.05.

Abbreviations: BMI, body mass index; CGM, continuous glucose monitor; CI, confidence interval; HCL, hybrid closed-loop; MDI, multiple daily injections; OR, odds ratio; Ref, reference.

**Table 3:** Multivariable analyses of associations between patient characteristics and annual visits with a registered dietitian in children with type 1 diabetes.

Multivariable analysis between patient characteristics (explanatory) and RD follow-up (outcome)*		
Characteristic	OR [95% CI]	*p*-Value
Age (years)	0.97 [0.94–0.99]	**0.004**
Sex		
Male	Ref	Ref
Female	0.98 [0.84–1.15]	0.833
Race and ethnicity		
White, non-Hispanic	Ref	Ref
Black, non-Hispanic	1.39 [0.96–2.00]	0.084
Hispanic	1.13 [0.83–1.53]	0.432
Asian, non-Hispanic	1.05 [0.59–1.88]	0.868
Multiracial, non-Hispanic	1.00 [0.58–1.73]	0.988
Another, non-Hispanic	1.14 [0.82–1.57]	0.431
Primary language		
English	Ref	Ref
Spanish	1.91 [0.68–5.35]	0.216
Other	0.61 [0.27–1.36]	0.224
Need for interpreter		
No	Ref	Ref
Yes	1.08 [0.43–2.68]	0.873
Insurance type		
Private	Ref	Ref
Public	1.16 [0.96–1.40]	0.135
Living in low-income zip codes		
No	Ref	Ref
Yes	1.03 [0.81–1.31]	0.873
Living in zip codes with lower educational attainment		
No	Ref	Ref
Yes	0.98 [0.77–1.25]	0.890
Diabetes duration (years)	0.85 [0.82–0.88]	**<0.001**
CGM use		
No	Ref	Ref
Yes	1.13 [0.94–1.37]	0.200
Mode of insulin delivery		
MDI	Ref	Ref
Insulin pump	1.01 [0.84–1.22]	0.901
HCL system	1.04 [0.80 to 1.34]	0.787
Calendar year		
2018	Ref	Ref
2019	0.90 [0.76–1.08]	0.265
2020	0.98 [0.81–1.19]	0.863
2021	0.85 [0.70–1.04]	0.110
2022	0.92 [0.74–1.13]	0.417
Analyses between RD follow-up (explanatory) and clinical characteristics within the subsequent year (outcome)		
Clinical characteristics	Coefficient [95% CI]	*p*-Value
Hemoglobin A1c (%)^†^	−0.09 [−0.16 to −0.01]	**0.029**
(mmol/mol)	(−0.93 [−1.77 to −0.09])	
BMI *z*-score^†^	0.01 [−0.02 to 0.04]	0.512

*Note*: Bold values are statistically significant associations, *p*<0.05.

Abbreviations: BMI, body mass index; CGM, continuous glucose monitor; CI, confidence interval; HCL, hybrid closed-loop; MDI, multiple daily injections; OR, odds ratio; RD, registered dietitian; Ref, reference.

* Analysis was adjusted for repeated measures and included all of the explanatory variables listed.

† Analyses were adjusted for repeated measures and controlled for age, sex, race and ethnicity, diabetes duration, CGM use, and mode of insulin delivery.

## Data Availability

The datasets generated during and analyzed in the current study and the analysis codes used are available from the corresponding author upon reasonable request.
