# Supplementary material for: Patient Characteristics Associated With Annual Nutrition Visits in Children With Type 1 Diabetes
Source: Pediatr Diabetes. 2025 Mar 28;2025:4108685. doi: 10.1155/pedi/4108685 (PMC12047747; doi:10.1155/pedi/4108685)
Supplement: Supplementary file 1 — Supporting Information 1 Table S1 summarizes detailed sociodemographic, diabetes care‐related, and clinical characteristics of children with type 1 diabetes by year subset by race and ethnicity. [file PEDI-2025-4108685-s001.docx]

**Supplemental Table S1. Characteristics of Established Pediatric Patients with Type 1 Diabetes by Year Subset by Race and Ethnicity**

| Characteristic | Data by Year (Observed Mean±SD or Percentage) | | | | |
| --- | --- | --- | --- | --- | --- |
|  | **2018** | **2019** | **2020** | **2021** | **2022** |
| Total patients  *White, non-Hispanic*  *Black, non-Hispanic*  *Hispanic* | 1206  *934*  *51*  *105* | 1193  *912*  *51*  *111* | 1181  *894*  *46*  *114* | 1226  *914*  *58*  *118* | 1228  *899*  *57*  *126* |
| Sociodemographic Characteristics |  |  |  |  |  |
| Age (years)  *White, non-Hispanic*  *Black, non-Hispanic*  *Hispanic* | 13.7±3.5  *13.8±3.5*  *13.9±3.3*  *13.6±3.4* | 13.7±3.6  *13.8±3.5*  *13.5±3.4*  *13.2±3.7* | 13.6±3.7  *13.7±3.7*  *13.3±3.1*  *13.0±3.6* | 13.5±3.7  *13.7±3.7*  *12.8±3.4*  *12.7±3.8* | 13.4±3.7  *13.5±3.8*  *13.1±3.2*  *13.0±3.4* |
| Female sex  *White, non-Hispanic*  *Black, non-Hispanic*  *Hispanic* | 48.8%  *48.4%*  *62.7%*  *44.8%* | 48.6%  *47.8%*  *70.6%*  *44.1%* | 50.0%  *49.7%*  *60.9%*  *47.4%* | 49.3%  *48.4%*  *63.8%*  *48.3%* | 47.3%  *46.5%*  *63.2%*  *45.2%* |
| Primary language^‡^ |  |  |  |  |  |
| English  *White, non-Hispanic*  *Black, non-Hispanic*  *Hispanic* | 96.6%  *99.4%*  *86.3%*  *80.0%* | 96.6%  *99.3%*  *84.3%*  *79.3%* | 96.0%  *99.2%*  *87.0%*  *78.1%* | 95.7%  *99.0%*  *87.9%*  *77.1%* | 95.6%  *98.8%*  *89.5%*  *76.2%* |
| Spanish  *White, non-Hispanic*  *Black, non-Hispanic*  *Hispanic* | 1.7%  *0.0%*  *2.0%*  *16.2%* | 1.8%  *0.0%*  *2.0%*  *17.1%* | 2.1%  *0.0%*  *0.0%*  *18.4%* | 2.2%  *0.0%*  *0.0%*  *19.5%* | 2.3%  *0.0%*  *0.0%*  *20.6%* |
| Other  *White, non-Hispanic*  *Black, non-Hispanic*  *Hispanic* | 1.7%  *0.6%*  *11.8%*  *3.8%* | 1.6%  *0.7%*  *13.7%*  *3.6%* | 1.9%  *0.8%*  *13.0%*  *3.5%* | 2.1%  *1.0%*  *12.1%*  *3.4%* | 2.1%  *1.2%*  *10.5%*  *3.2%* |
| Need for interpreter  *White, non-Hispanic*  *Black, non-Hispanic*  *Hispanic* | 2.4%  *0.2%*  *11.8%*  *17.1%* | 2.8%  *0.2%*  *11.8%*  *20.7%* | 3.0%  *0.2%*  *8.7%*  *21.1%* | 3.2%  *0.3%*  *8.6%*  *21.2%* | 3.5%  *0.6%*  *7.0%*  *22.2%* |
| Public insurance  *White, non-Hispanic*  *Black, non-Hispanic*  *Hispanic* | 23.6%  *15.3%*  *66.7%*  *64.8%* | 24.0%  *15.5%*  *62.7%*  *64.9%* | 24.8%  *16.1%*  *58.7%*  *65.8%* | 26.9%  *17.2%*  *70.7%*  *67.8%* | 28.8%  *19.2%*  *73.7%*  *67.5%* |
| Living in low-income zip codes^†^  *White, non-Hispanic*  *Black, non-Hispanic*  *Hispanic* | 24.5%  *16.6%*  *80.0%*  *61.0%* | 25.1%  *16.5%*  *78.0%*  *64.9%* | 24.7%  *15.6%*  *66.7%*  *68.4%* | 25.1%  *15.7%*  *70.7%*  *65.3%* | 24.5%  *15.0%*  *73.7%*  *62.7%* |
| Living in zip codes with lower educational attainment^†^  *White, non-Hispanic*  *Black, non-Hispanic*  *Hispanic* | 25.1%  *18.8% 72.0%*  *55.2%* | 24.7%  *17.5%*  *72.0%*  *53.2%* | 25.0%  *18.1%*  *62.2%*  *58.8%* | 25.1%  *18.0%*  *67.2%*  *53.4%* | 25.6%  *18.2%*  *66.7%*  *53.2%* |
| Diabetes Care-related Characteristics |  |  |  |  |  |
| Diabetes duration (years)  *White, non-Hispanic*  *Black, non-Hispanic*  *Hispanic* | 6.0±3.7  *6.1±3.8*  *5.2±3.0*  *5.9±3.8* | 5.9±3.6  *6.0±3.8*  *5.0±2.8*  *5.7±3.7* | 5.7±3.7  *5.9±3.7*  *5.0±2.8*  *5.5±3.8* | 5.6±3.6  *5.8±3.7*  *4.5±2.8*  *5.4±3.7* | 5.5±3.4  *5.5±3.4*  *5.1±3.0*  *5.3±3.6* |
| CGM use^§^  *White, non-Hispanic*  *Black, non-Hispanic*  *Hispanic* | 52.3%  *56.7%*  *23.5%*  *31.4%* | 71.3%  *76.6%*^*^  *41.2%*^*^  *45.9%*^*^ | 78.4%  *82.2%*^*^  *52.2%*^*^  *59.6%*^*^ | 84.7%  *87.5%*^*^  *70.7%*^*^  *70.3%*^*^ | 89.9%  *92.9%*^*^  *77.2%*^*^  *77.0%*^*^ |
| Mode of insulin delivery^‡§^ |  |  |  |  |  |
| MDI  *White, non-Hispanic*  *Black, non-Hispanic*  *Hispanic* | 38.9%  *31.8%*  *94.1%*  *62.9%* | 38.2%  *31.1%*^*^  *84.3%*  *61.3%* | 39.7%  *32.0%*  *87.0%*  *67.5%* | 41.0%  *34.1%*  *86.2%*  *63.6%* | 36.9%  *29.6%*^*^  *82.5%*  *61.9%* |
| Insulin pump  *White, non-Hispanic*  *Black, non-Hispanic*  *Hispanic* | 58.0%  *64.6%*  *5.9%*  *37.1%* | 58.4%  *64.8%*  *15.7%*  *38.7%* | 48.4%  *54.3%*^*^  *13.0%*  *23.7%*^*^ | 41.0%  *45.2%*^*^  *13.8%*  *26.3%*^*^ | 26.3%  *27.5%*^*^  *12.3%*  *18.3%*^*^ |
| HCL system  *White, non-Hispanic*  *Black, non-Hispanic*  *Hispanic* | 3.1%  *3.6%*  *0.0%*  *0.0%* | 3.4%  *4.1%*  *0.0%*  *0.0%* | 11.9%  *13.8%*^*^  *0.0%*  *8.8%*^\|\|^ | 17.9%  *20.7%*^*^  *0.0%*  *10.2%*^\|\|^ | 36.8%  *42.9%*^*^  *5.3%*  *19.8%* |
| Clinical Characteristics |  |  |  |  |  |
| HbA1c (%)^¶^  [mmol/mol]  *White, non-Hispanic*  *Black, non-Hispanic*  *Hispanic* | 8.5±1.5  [69±16]  *8.3±1.4*  *[68±15]*  *9.6±1.8*  *[81±19]*  *9.0±1.8*  *[75±20]* | 8.3±1.6  [67±18]  *8.1±1.4*^*^  *[65±16]*  *10.3±2.5*^*^  *[89±27]*  *8.8±1.9*  *[73±21]* | 8.2±1.6  [67±17]  *8.0±1.4*  *[64±15]*  *9.7±2.4*  *[83±26]*  *8.9±2.0*  *[74±22]* | 7.9±1.6  [63±18]  *7.7±1.4*^*^  *[60±15]*  *9.7±2.4*^*^  *[82±26]*  *8.7±1.8*  *[72±20]* | 7.8±1.6  [61±18]  *7.5±1.4*^*^  *[59±15]*  *9.4±2.4*  *[79±26]*  *8.6±2.0*  *[70±22]* |
| BMI z-score^¶^  *White, non-Hispanic*  *Black, non-Hispanic*  *Hispanic* | 0.63±0.94  *0.64±0.91*  *0.78±1.17*  *0.80±1.02* | 0.65±0.95  *0.65±0.92*^*^  *0.58±1.18*  *0.84±1.01* | 0.69±0.95  *0.68±0.92*  *0.55±1.16*  *0.86±1.08* | 0.70±0.95  *0.68±0.93*^*^  *0.58±1.11*  *0.92±1.07* | 0.71±0.96  *0.70±0.93*^*^  *0.55±1.15*  *0.85±1.03* |

^*^ Values in these years differed significantly from 2018, *P* < 0.05.

^†^ Missing data was not included in the denominator.

^‡^ Totals may not sum up to 100% due to rounding.

**^§^** Although observed values are reported, comparative analyses across years were adjusted for repeated measures and controlled for age, sex, and diabetes duration.

^||^ Values in these years differed significantly from 2022, *P* < 0.05.

^¶^ Although observed values are reported, comparative analyses across years were adjusted for repeated measures and controlled for age, sex, diabetes duration, CGM use, and mode of insulin delivery.

*Abbreviations:* BMI, body mass index; CGM, continuous glucose monitor; HbA1c, hemoglobin A1c; HCL, hybrid closed-loop; MDI, multiple daily injections; RD, registered dietitian; SD, standard deviation.
