# Supplementary material for: Patient Characteristics Associated With Annual Nutrition Visits in Children With Type 1 Diabetes
Source: Pediatr Diabetes. 2025 Mar 28;2025:4108685. doi: 10.1155/pedi/4108685 (PMC12047747; doi:10.1155/pedi/4108685)
Supplement: Supplementary file 2 — Supporting Information 2 Table S2 summarizes associations between patient characteristics and annual visits with a registered dietitian in children with type 1 diabetes subset by race and ethnicity (Table S2a: White, non‐Hispanic patients; Table S2b: Black, non‐Hispanic patients; and Table S2c: Hispanic patients). [file PEDI-2025-4108685-s002.docx]

**Supplemental Table S2a.** **Associations Between Patient Characteristics and Annual Visits with a Registered Dietitian in Children with Type 1 Diabetes among White, non-Hispanic Patients**

| **Multivariable analysis between patient characteristics (explanatory) and RD follow-up (outcome)^*^** | | |
| --- | --- | --- |
| **Characteristic** | **OR (95% CI)** | ***P* value** |
| Age (years) | 0.96 (0.94 to 0.99) | **0.004** |
| Sex |  |  |
| *Male* | REF | REF |
| *Female* | 0.96 (0.80 to 1.16) | 0.699 |
| Primary language |  |  |
| *English* | REF | REF |
| *Other* | 0.96 (0.34 to 2.70) | 0.936 |
| Need for interpreter |  |  |
| *No* | REF | REF |
| *Yes* | 0.17 (0.02 to 1.21) | 0.078 |
| Insurance type |  |  |
| *Private* | REF | REF |
| *Public* | 1.10 (0.87 to 1.39) | 0.427 |
| Living in low-income zip codes |  |  |
| *No* | REF | REF |
| *Yes* | 1.09 (0.81 to 1.48) | 0.573 |
| Living in zip codes with lower educational attainment |  |  |
| *No* | REF | REF |
| *Yes* | 0.87 (0.64 to 1.18) | 0.367 |
| Diabetes duration (years) | 0.85 (0.82 to 0.89) | **<0.001** |
| CGM use |  |  |
| *No* | REF | REF |
| *Yes* | 1.14 (0.90 to 1.46) | 0.274 |
| Mode of insulin delivery |  |  |
| *MDI* | REF | REF |
| *Insulin pump* | 0.96 (0.78 to 1.18) | 0.667 |
| *HCL system* | 0.99 (0.74 to 1.31) | 0.936 |
| Calendar year |  |  |
| *2018* | REF | REF |
| *2019* | 0.95 (0.77 to 1.16) | 0.614 |
| *2020* | 1.09 (0.87 to 1.36) | 0.451 |
| *2021* | 0.95 (0.76 to 1.19) | 0.665 |
| *2022* | 1.05 (0.82 to 1.35) | 0.698 |
| **Analyses between RD follow-up (explanatory) and clinical characteristics within the subsequent year (outcome)** | | |
| **Clinical Characteristics** | **Coefficient (95% CI)** | ***P* value** |
| Hemoglobin A1c (%)**^†^**  [mmol/mol] | -0.09 (-0.17 to -0.00)  [-0.96 (-1.87 to -0.05)] | **0.038** |
| BMI z-score**^†^** | 0.02 (-0.02 to 0.05) | 0.350 |

^*^ Analysis was adjusted for repeated measures and included all of the explanatory variables listed.

**^†^** Analyses were adjusted for repeated measures and controlled for age, sex, diabetes duration, CGM use, and mode of insulin delivery.

*Abbreviations:* BMI, body mass index; CGM, continuous glucose monitor; CI, confidence interval; HCL, hybrid closed-loop; MDI, multiple daily injections; OR, odds ratio; RD, registered dietitian; REF, reference.

**Supplemental Table S2b.** **Associations Between Patient Characteristics and Annual Visits with a Registered Dietitian in Children with Type 1 Diabetes among Black, non-Hispanic Patients**

| **Multivariable analysis between patient characteristics (explanatory) and RD follow-up (outcome)^*^** | | | |
| --- | --- | --- | --- |
| **Characteristic** | **OR (95% CI)** | ***P* value** |  |
| Age (years) | 1.03 (0.90 to 1.18) | 0.647 |  |
| Sex |  |  |  |
| *Male* | REF | REF |  |
| *Female* | 0.76 (0.36 to 1.60) | 0.470 |  |
| Language |  |  |  |
| *English* | REF | REF |  |
| *Other* | 0.28 (0.07 to 1.07) | 0.062 |  |
| Need for interpreter |  |  |  |
| *No* | REF | REF |  |
| *Yes* | 2.27 (0.30 to 17.08) | 0.427 |  |
| Insurance type |  |  |  |
| *Private* | REF | REF |  |
| *Public* | 1.86 (0.92 to 3.76) | 0.084 |  |
| Living in low-income zip codes |  |  |  |
| *No* | REF | REF |  |
| *Yes* | 1.20 (0.37 to 3.92) | 0.765 |  |
| Living in zip codes with lower educational attainment |  |  |  |
| *No* | REF | REF |  |
| *Yes* | 0.99 (0.34 to 2.90) | 0.984 |  |
| Diabetes duration (years) | 0.81 (0.66 to 0.98) | **0.032** |  |
| CGM use |  |  |  |
| *No* | REF | REF |  |
| *Yes* | 1.13 (0.50 to 2.57) | 0.771 |  |
| Mode of insulin delivery |  |  |  |
| *MDI* | REF | REF |  |
| *Insulin pump* | 1.60 (0.53 to 4.83) | 0.401 |  |
| *HCL system* | 0.85 (0.05 to 13.88) | 0.910 |  |
| Calendar year |  |  |  |
| *2018* | REF | REF |  |
| *2019* | 0.58 (0.21 to 1.63) | 0.300 |  |
| *2020* | 1.45 (0.56 to 3.75) | 0.447 |  |
| *2021* | 1.00 (0.39 to 2.58) | 0.996 |  |
| *2022* | 0.80 (0.32 to 1.98) | 0.627 |  |
| **Analyses between RD follow-up (explanatory) and clinical characteristics within the subsequent year (outcome)** | | | |
| **Clinical Characteristics** | **Coefficient (95% CI)** | ***P* value** |  |
| Hemoglobin A1c (%)**^†^**  [mmol/mol] | 0.22 (-0.26 to 0.71)  [2.42 (-2.87 to 7.72)] | 0.370 |  |
| BMI z-score**^†^** | 0.24 (-0.09 to 0.57) | 0.155 |  |

^*^ Analysis was adjusted for repeated measures and included all of the explanatory variables listed.

**^†^** Analyses were adjusted for repeated measures and controlled for age, sex, diabetes duration, CGM use, and mode of insulin delivery.

*Abbreviations:* BMI, body mass index; CGM, continuous glucose monitor; CI, confidence interval; HCL, hybrid closed-loop; MDI, multiple daily injections; OR, odds ratio; RD, registered dietitian; REF, reference.

**Supplemental Table S2c.** **Associations Between Patient Characteristics and Annual Visits with a Registered Dietitian in Children with Type 1 Diabetes among Hispanic Patients**

| **Multivariable analysis between patient characteristics (explanatory) and RD follow-up (outcome)^*^** | | |
| --- | --- | --- |
| **Characteristic** | **OR (95% CI)** | ***P* value** |
| Age (years) | 0.97 (0.90 to 1.04) | 0.414 |
| Sex |  |  |
| *Male* | REF | REF |
| *Female* | 1.22 (0.75 to 1.96) | 0.424 |
| Language |  |  |
| *English* | REF | REF |
| *Spanish* | 0.73 (0.24 to 2.19) | 0.569 |
| *Other* | 0.27 (0.07 to 1.05) | 0.058 |
| Need for interpreter |  |  |
| *No* | REF | REF |
| *Yes* | 2.93 (1.01 to 8.54) | **0.048** |
| Insurance type |  |  |
| *Private* | REF | REF |
| *Public* | 1.08 (0.61 to 1.92) | 0.794 |
| Living in low-income zip codes |  |  |
| *No* | REF | REF |
| *Yes* | 0.84 (0.46 to 1.55) | 0.585 |
| Living in zip codes with lower educational attainment |  |  |
| *No* | REF | REF |
| *Yes* | 1.77 (1.00 to 3.13) | **0.049** |
| Diabetes duration (years) | 0.87 (0.80 to 0.95) | **0.003** |
| CGM use |  |  |
| *No* | REF | REF |
| *Yes* | 1.15 (0.76 to 1.74) | 0.500 |
| Mode of insulin delivery |  |  |
| *MDI* | REF | REF |
| *Insulin pump* | 1.37 (0.82 to 2.29) | 0.229 |
| *HCL system* | 0.59 (0.21 to 1.65) | 0.316 |
| Calendar year |  |  |
| *2018* | REF | REF |
| *2019* | 0.65 (0.38 to 1.12) | 0.123 |
| *2020* | 0.67 (0.39 to 1.14) | 0.139 |
| *2021* | 0.55 (0.31 to 0.97) | **0.040** |
| *2022* | 0.63 (0.35 to 1.13) | 0.122 |
| **Analyses between RD follow-up (explanatory) and clinical characteristics within the subsequent year (outcome)** | | |
| **Clinical Characteristics** | **Coefficient (95% CI)** | ***P* value** |
| Hemoglobin A1c (%)**^†^**  [mmol/mol] | -0.27 (-0.52 to -0.02)  [-2.95 (-5.67 to -0.23)] | **0.034** |
| BMI z-score**^†^** | 0.00 (-0.11 to 0.12) | 0.951 |

^*^ Analysis was adjusted for repeated measures and included all of the explanatory variables listed.

**^†^** Analyses were adjusted for repeated measures and controlled for age, sex, diabetes duration, CGM use, and mode of insulin delivery.

*Abbreviations:* BMI, body mass index; CGM, continuous glucose monitor; CI, confidence interval; HCL, hybrid closed-loop; MDI, multiple daily injections; OR, odds ratio; RD, registered dietitian; REF, reference.
